# Observed trends in the magnitude and persistence of monthly temperature variability

**DOI:** 10.1038/s41598-017-06382-x

**Published:** 2017-07-19

**Authors:** Timothy M. Lenton, Vasilis Dakos, Sebastian Bathiany, Marten Scheffer

**Affiliations:** 1 Earth System Science Group, College of Life and Environmental Sciences, University of Exeter, Exeter, EX4 4QE UK; 2 Institute of Integrative Biology, Center for Adaptation to a Changing Environment, ETH Zurich, Zurich, Switzerland; 3 Institut des Sciences de l’Evolution de Montpellier (ISEM), BioDICée team, Université de Montpellier, CNRS, IRD, EPHE, place Eugène Bataillon, UMR 5554, CC065, Montpellier, 34095 Montpellier Cedex 05 France; 4 Department of Environmental Sciences, Wageningen University, P.O. Box 47, NL-6700 AA Wageningen, The Netherlands

## Abstract

Climate variability is critically important for nature and society, especially if it increases in amplitude and/or fluctuations become more persistent. However, the issues of whether climate variability is changing, and if so, whether this is due to anthropogenic forcing, are subjects of ongoing debate. Increases in the amplitude and persistence of temperature fluctuations have been detected in some regions, e.g. the North Pacific, but there is no agreed global signal. Here we systematically scan monthly surface temperature indices and spatial datasets to look for trends in variance and autocorrelation (persistence). We show that monthly temperature variability and autocorrelation increased over 1957–2002 across large parts of the North Pacific, North Atlantic, North America and the Mediterranean. Furthermore, (multi)decadal internal climate variability appears to influence trends in monthly temperature variability and autocorrelation. Historically-forced climate models do not reproduce the observed trends in temperature variance and autocorrelation, consistent with the models poorly capturing (multi)decadal internal climate variability. Based on a review of established spatial correlations and corresponding mechanistic ‘teleconnections’ we hypothesise that observed slowing down of sea surface temperature variability contributed to observed increases in land temperature variability and autocorrelation, which in turn contributed to persistent droughts in North America and the Mediterranean.

## Introduction

While the magnitude of long term change in the climate is important, society and ecosystems are particularly sensitive to climate variability and its extremes. In this study we set out to explore observed trends in the magnitude and persistence of climate variability. These are of interest from both a social and an ecological perspective. The magnitude of climate variability clearly affects the magnitude of social^1^ and ecological^2^ impacts, but so too can the persistence (or time correlation) of variability. This is true not just because a longer event accumulates more impacts, but also because it can have impacts greater than the sum of its parts. For instance, a long heat wave can have greater impacts on human mortality than the sum of individual hot days^3^, and multi-year droughts can have greater agricultural economic impacts than the sum of individual dry years^4^. Persistent drought and its impact on agriculture and food prices can in turn contribute to social unrest^5^. In ecology, time-correlated disturbance affects the magnitude of ecosystem change^6, 7^ and the possibility that ecosystems are pushed across tipping points for irreversible change^7, 8^. Furthermore, trends in variance and temporal correlation can help to diagnose important underlying changes in the ‘resilience’ of parts of the climate system – meaning the strength of restoring negative feedbacks. In particular, a combined increase in autocorrelation and variance are a signal that restoring negative feedbacks in a system are getting weaker (i.e. declining resilience), and they may also indicate (in extreme cases) an approach to a bifurcation-type ‘tipping point’^9–11^.

Existing work has sought to address whether temperature variability is changing at the global scale due to climate change^12–14^, but with no firm conclusion as yet. Inter-annual temperature variability has increased in some regions but decreased in others^13^. However, several published calculations of changes in variability are biased by normalising to a fixed reference interval^15^. Trends in the persistence of temperature variability, as measured by e.g. changing lag-1 autocorrelation, are generally less studied. One exception is recent work showing a marked slowing down of inter-monthly sea surface temperature variability in the North Pacific^7^, comprising increases in both autocorrelation and variance. Here we seek to extend previous global analyses that examined variance in annual mean temperature data^12, 13^, by studying monthly temperature data for trends in lag-1 autocorrelation as well as variance (measured as standard deviation). This also extends previous regional analysis^7^, of monthly sea surface temperature data and the Pacific Decadal Oscillation index, to other regions and climate indices. Wherever possible we seek to avoid the biasing caused by normalising to a fixed reference interval^15^.

Our analysis focuses on monthly mean temperature datasets with the seasonal cycle and long-term warming trend removed (see Methods). The longest temperature records come from regions with early weather stations and early ship-borne thermometer measurements – with the best data coverage over the oceans in the North Atlantic and North Pacific where there have been regular ship routes, especially since the 1950s. Here we focus on the well-characterised interval of the ERA-40 atmospheric reanalysis^16^, 1957–2002 (a focus of previous global inter-annual temperature analysis)^13^. Several different temperature reconstructions exist over this interval and we seek to compare these to check the robustness of our results. We pay particular attention to Northern Hemisphere ocean regions, corresponding climate indices, and those parts of the land surface they are known to influence.

The paper is organised as follows: First we examine trends in autocorrelation and variance in Northern Hemisphere climate indices. Then we consider whether existing historically forced model runs reproduce these trends. Next we analyse the spatial pattern of observed autocorrelation and variance trends in monthly temperature data. Finally we review existing studies in an attempt to mechanistically link some observed trends in the persistence of ocean surface temperature variability to observed extreme events on land.

## Results

### Northern Hemisphere climate indices

We started by analysing climate indices that have been derived from the dominant spatial patterns of variability in Northern Hemisphere sea surface temperature data, specifically the Pacific Decadal Oscillation (PDO), the Atlantic Multi-decadal Oscillation (AMO), and the Atlantic Tripole. The latter represents the lagged response of ocean surface temperatures to the dominant mode of North Atlantic pressure variability, the North Atlantic Oscillation (NAO), which we also analyse for completeness (noting that atmospheric pressure variability has far less memory – i.e. much lower autocorrelation – than sea surface temperatures).

**Figure 1:**
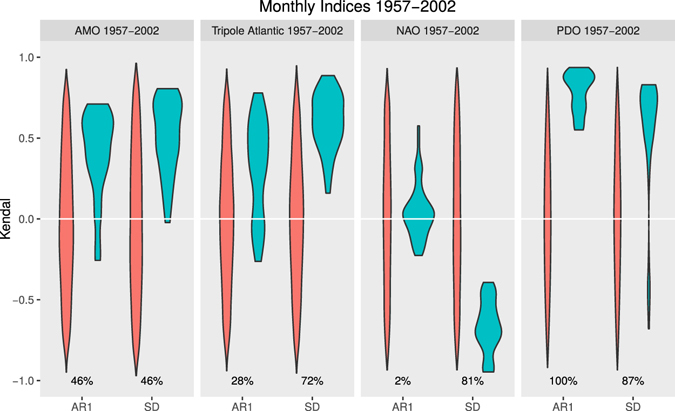
Autocorrelation and variance trends in climate indices. For the ERA-40 interval (09/1957-08/2002). Distribution of Kendall τ trends in AR(1) and standard deviation for all combinations of sliding window and bandwidth size in observational climate indices (blue-green) and null models (red; from 1000 time-series with same frequency spectrum) (see Methods sections Sensitivity analysis, Significance testing). The percentages represent the fraction of results from the observational indices that are significantly different to the null models (*p* = 0.1 two tailed).

We find consistent trends in the character of fluctuations in the chosen climate indices (Fig. 1). Standardising the interval of comparison to the ERA-40 reanalysis^16^ interval 1957–2002 (Fig. 1), relative to longer original datasets (Figs S1–S5), has little qualitative effect on the results (compare Figs 1 and S5), with the exception that variance in the PDO index increases more markedly over 1957–2002 than over 1948–2016 (as an interval of high initial variance is removed). The signs of the autocorrelation and variance trends are generally robust to choice of filtering bandwidth and sliding window size used in the analysis, although their magnitude varies (Figs 1 and S5–6). Over 1957–2002 (Figs 1 and S6 and Table 1), the PDO index shows a strong increase in autocorrelation (median Kendall τ = 0.84) and variance (τ = 0.68), as examined previously^7^. The AMO index shows increases in both autocorrelation (τ = 0.47) and variance (τ = 0.53). The Atlantic tripole index also shows increases in both autocorrelation (τ = 0.36) and variance (τ = 0.61), whereas the NAO index shows no overall trend in autocorrelation (τ = 0.04) and a strong decline in variance (τ = −0.68).

**Table 1:** Autocorrelation and variance trends in observational climate indices (1957–2002).

Index	AR1 median	(5,95)		SD median	(5,95)	
AMO	0.47	−0.21	0.69	0.53	0.09	0.79
Atlantic tripole	0.36	−0.15	0.72	0.61	0.29	0.85
NAO	0.04	−0.17	0.35	−0.68	−0.94	−0.44
PDO	0.84	0.56	0.92	0.68	−0.47	0.83

Trends expressed as Kendall τ values. Medians as well as 5 and 95 percentiles from the estimated distributions (varying filtering bandwidth and sliding window length) are reported.

To establish whether these trends in autocorrelation and variance are significant we test against a null model of 1000 time-series of surrogate data with the same frequency spectrum (see Methods). Over 1957–2002 (Fig. 1), the increase in autocorrelation in the observed PDO index is the most significant, with 100% of the trends from different filtering bandwidth-sliding window combinations significantly different to those from the null model. 91% of the increasing variance trends in the PDO index are significant. For the increases in autocorrelation and variance in the AMO index, 46% and 56% respectively of the trends are significant. For the increases in autocorrelation and variance in the Atlantic Tripole index 30% and 67% respectively of the trends are significant. For the decline in variance in the NAO index 87% of the trends are significant.

**Figure 2:**
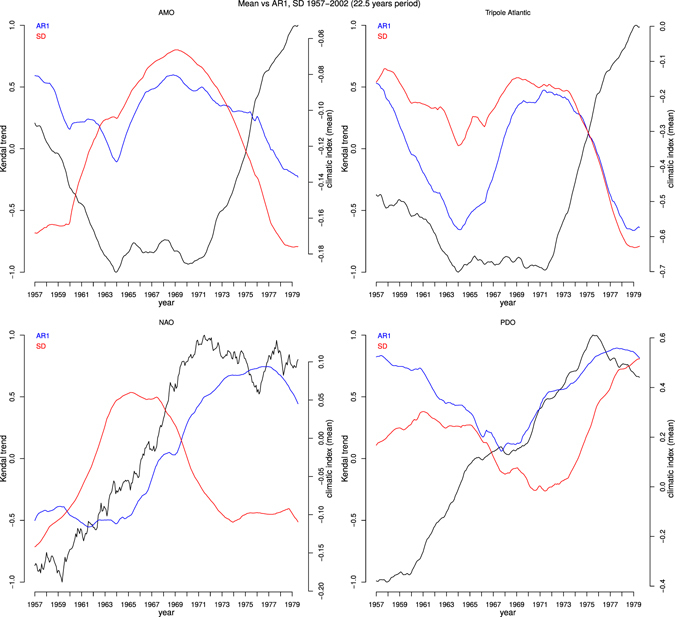
Decadal variability in autocorrelation and variance trends in climate indices. For 22.5 year intervals (within 1957–2002) analysed with filtering bandwidth and sliding window length of half the interval (11 years 3 months). Also plotted is the mean value of the climate index over the same 22.5 year intervals. (top left) AMO, (top right) Atlantic Tripole, (bottom left) NAO, (bottom right) PDO.

Interestingly, when shortening the interval in which autocorrelation and variance trends are calculated to 22.5 years (half the 1957–2002 series) and varying the timing of that 22.5 year interval, the sign of calculated autocorrelation and variance trends can vary (Figs 2, S7 and S8). The PDO autocorrelation trend is strongly positive initially, weakens then strengthens again, whereas the PDO variance trend starts weakly positive, switches to weakly negative then recovers to strongly positive. The AMO autocorrelation trend is generally positive but weak and fluctuates in strength with a ~15 year period. The AMO variance trends switches from strongly negative to strongly positive then back to strongly negative, in antiphase with the index itself – indicating that an interval of negative AMO is associated with increasing short-term variability of North Atlantic SST fluctuations. The Atlantic Tripole autocorrelation trend switches between positive and negative on a ~15 year period (somewhat similar to AMO autocorrelation trends). The Atlantic Tripole variance trend is more predominantly positive but follows a similar pattern. The NAO autocorrelation trend switches from negative to positive roughly in phase with the overall negative to positive shift in the index itself, whereas the variance trend is initially strongly negative, switches to positive then returns to strongly negative.

Whilst some of the (multi)decadal variability in autocorrelation and variance trends could occur by chance in a red-noise system (due to finite sampling), the results (Figs 2, S7 and S8) also suggest that the low-frequency internal modes of climate variability themselves may cause changes in the autocorrelation and variance of shorter-term fluctuations. This seems mechanistically plausible if, for example, a shift within a mode of variability is accompanied by systematic regional changes in wind strength and/or ocean mixed layer depth, which affect the decay rate of sea surface temperature fluctuations^17^. Despite the decadal variability in autocorrelation and variance trends there are still overall positive trends over the full 1957–2002 interval, especially in the PDO index (which also shows increasing autocorrelation and variance over longer intervals)^7^, leaving open the possibility of a forced component to such longer term trends.

### Model historical simulations

**Figure 3:**
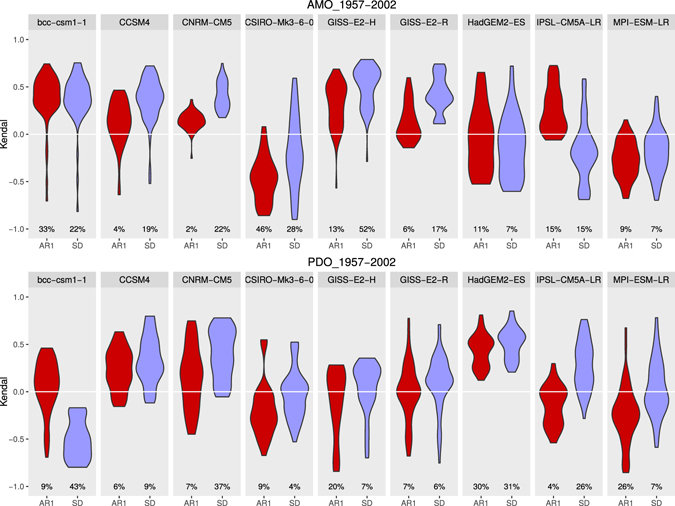
Modelled historical trends in autocorrelation and variance in the PDO and AMO indices. Results for nine models in the CMIP5 database under historical forcing (followed by RCP8.5) for the ERA-40 interval (09/1957-08/2002). Distribution of Kendall τ trends in AR(1) (red) and standard deviation (purple) for all combinations of sliding window and bandwidth size (see Methods section Sensitivity). The percentages represent the fraction of results from the modelled indices that are significantly different to null models (*p* = 0.1 two tailed).

To examine further whether any anthropogenic forced component is apparent in observed autocorrelation and variance trends, we analysed fluctuations in the AMO and PDO indices simulated by nine climate models from the CMIP5 database over the same historical interval 1957–2002 (see Methods). The results (Fig. 3 and Table 2) show generally weak and mixed autocorrelation and variance trends in the modelled AMO and PDO indices, as may be expected by chance (without a strong forced component). None of the models reproduce the strength of positive trend in autocorrelation in the observed AMO index (median τ = 0.47), although one model (GISS-E2-H) produces a comparable positive trend in variance (τ = 0.58) to the observations (τ = 0.53). None of the models reproduce the strength of positive trends in autocorrelation (τ = 0.84) and variance (τ = 0.68) seen in the observed PDO index, and only one model (HadGEM2-ES) produces robustly positive trends in both autocorrelation (τ = 0.48) and variance (τ = 0.54) in its modelled PDO index.

**Table 2:** Autocorrelation and variance trends in model simulations of the AMO and PDO climate indices (1957–2002).

Model	ΑΜΟ						PDO					
	AR1 median	(5,95)		SD median	(5,95)		AR1 median	(5,95)		SD median	(5,95)	
bcc-csm1-1	0.41	−0.16	0.65	0.37	0.01	0.71	0.10	−0.50	0.43	−0.52	−0.78	−0.19
CCSM4	0.14	−0.22	0.44	0.40	0.06	0.66	0.25	−0.11	0.58	0.32	−0.06	0.79
CNRM-CM5	0.15	0.01	0.27	0.37	0.22	0.62	0.19	−0.37	0.69	0.41	−0.03	0.71
CSIRO-Mk3-6-0	−0.48	−0.82	−0.06	−0.25	−0.84	0.45	−0.25	−0.53	0.53	0.00	−0.38	0.51
GISS-E2-H	0.35	−0.01	0.59	0.58	0.22	0.75	−0.01	−0.80	0.26	0.13	−0.54	0.33
GISS-E2-R	0.07	−0.11	0.50	0.40	0.12	0.73	0.01	−0.53	0.44	0.13	−0.47	0.46
HadGEM2-ES	−0.04	−0.49	0.56	−0.13	−0.55	0.39	0.48	0.20	0.70	0.54	0.26	0.77
IPSL-CM5A-LR	0.21	−0.02	0.66	−0.20	−0.65	0.42	−0.09	−0.46	0.14	0.30	−0.08	0.69
MPI-ESM-LR	−0.27	−0.61	0.01	−0.20	−0.56	0.22	−0.23	−0.77	0.19	−0.04	−0.44	0.52

Trends expressed as Kendall τ values. Medians as well as 5 and 95 percentiles from the estimated distributions (varying filtering bandwidth and sliding window length) are reported.

Thus, there is no consistent signal of an anthropogenic forced trend in either autocorrelation or variance in either the AMO or PDO as simulated across these model runs. This could be because the models fail to respond to forcing correctly. The models are known to be of varying quality in their ability to capture internal (multi)decadal variability and in no case would the timing of low-frequency variability be expected to match the real system because these are not data-assimilated model runs. Hence given the small number of model realisations, the results are consistent with the interpretation that historical trends in autocorrelation and variance in the climate indices are influenced by (multi)decadal internal variability of the climate system. The fact that there are significant long-term positive trends in autocorrelation and variance in the observations (Fig. 1), most strongly for the PDO index, but also in the AMO and Atlantic Tripole indices, leaves open the possibility that there is a forced component to these trends which the models are failing to capture.

### Spatial trends in persistence and variance

Having established that there have been some long-term trends in autocorrelation and variance of key Northern Hemisphere climate indices as well as (multi)decadal modulation of those trends, we considered the spatial pattern of trends in autocorrelation and variance. For this we analysed the HadCRUT4^18^ and GISTEMP^19^ surface temperature datasets and the atmospheric reanalysis dataset ERA-40^16^ again over the common interval 1957–2002. Whilst the two temperature datasets come as anomalies relative to a baseline period, which can bias estimates of variance outside of the reference period^15^, the reanalysis dataset comes in absolute values allowing us to remove a running mean and thus avoid biasing the variance estimates^15^.

**Figure 4:**
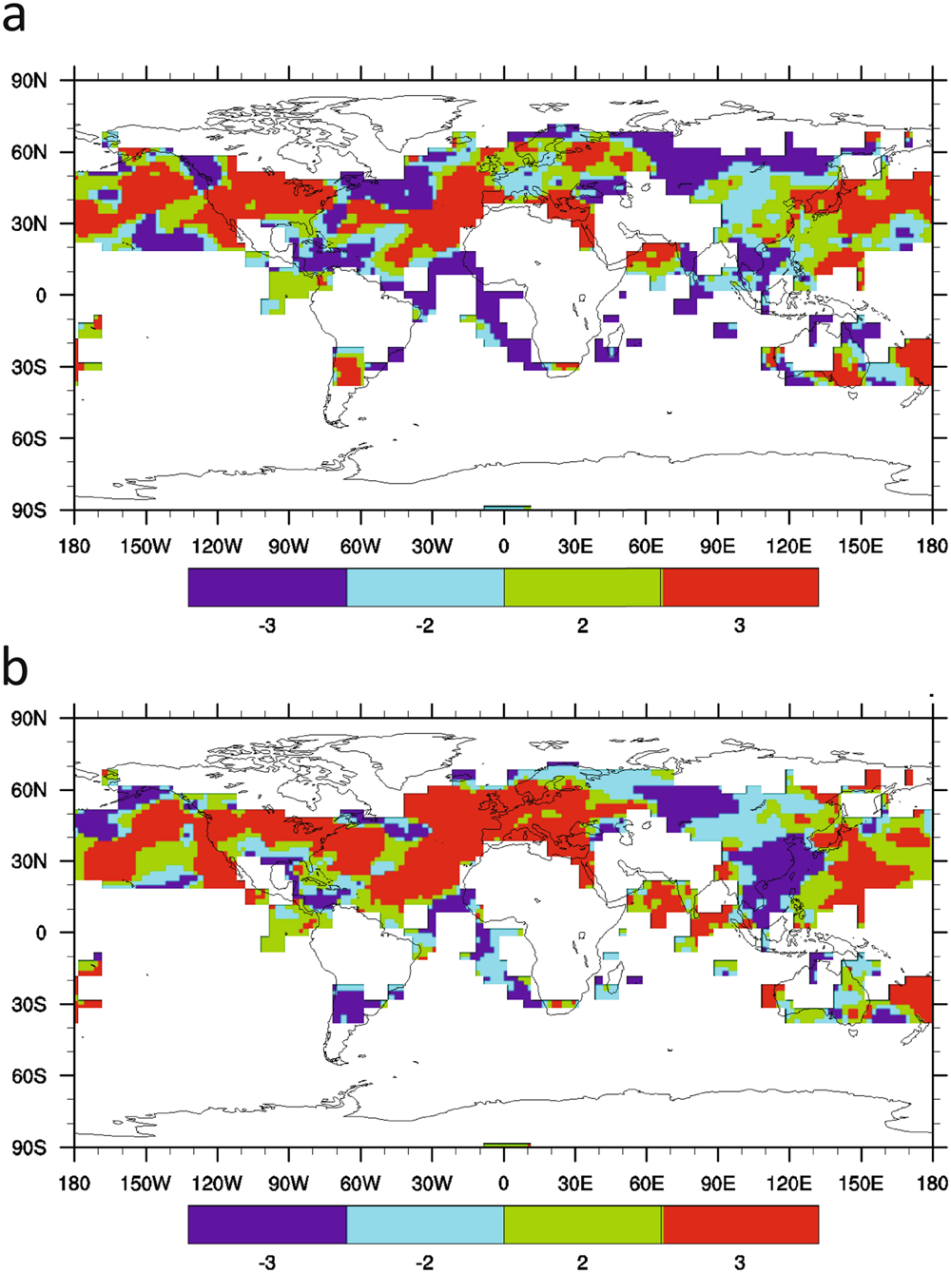
Consistency in autocorrelation and variance trends between temperature datasets. Monthly temperature datasets HadCRUT4, GISTEMP and ERA-40 in the interval 1957–2002, processed with filtering bandwidth 10 years and sliding window length 25 years. (**a**) AR(1). (**b**) Standard deviation. Red (3) indicates all 3 datasets agree on a positive trend, dark blue (−3) indicates all 3 datasets agree on a negative trend, green (2) indicates 2 datasets give a positive trend, 1 gives a negative trend, cyan (−2) indicates 2 datasets give a negative trend, 1 gives a positive trend. Maps were created using NCAR Command Language (Version 6.2.1) [Software]. (2014). Boulder, Colorado: UCAR/NCAR/CISL/TDD. http://dx.doi.org/10.5065/D6WD3XH5.

The analysis of spatial temperature data over 1957–2002 shows somewhat different global spatial patterns of trends for either autocorrelation or variance across different datasets, but also large regions of agreement (Fig. 4). Focusing on the HadCRUT4 regions (where there is good raw data coverage), the different datasets agree that autocorrelation increased across large parts of the North Pacific, the North Atlantic, North America, and the Mediterranean, and in the Arabian Sea. They agree that variance increased in broadly the same regions, with the increase in variance being more widespread (than increasing autocorrelation) across the North Atlantic and Europe. (The spatial pattern of change in inter-monthly temperature variance is also broadly similar to the previously published pattern of change in inter-annual temperature variability)^13^.

Within individual temperature datasets, there are thus typically consistent increases in both autocorrelation and variance across large parts of the North Pacific, North America, in a SW-NE band across the North Atlantic, and through the Mediterranean (Fig. S9). In other regions, e.g. much of Siberian Russia, there are consistent decreases in autocorrelation and variance. Within each dataset there are also cross-over regions with inconsistent trends in autocorrelation and variance. Trends in autocorrelation and variance are thus not related globally in a systematic way.

**Figure 5:**
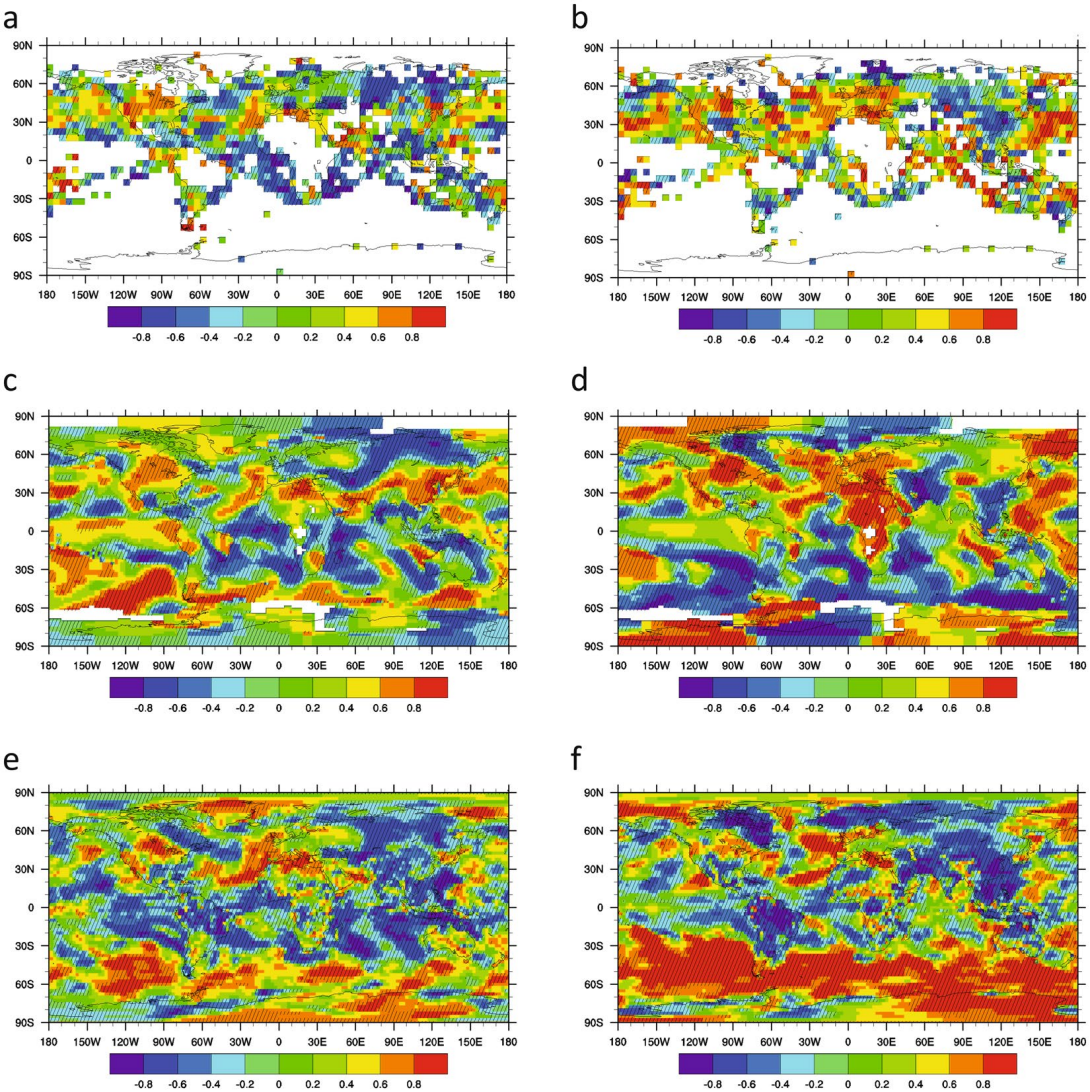
Trends in autocorrelation and variance in different temperature datasets 1957–2002. Monthly temperature datasets processed with filtering bandwidth 10 years and sliding window length 25 years: (**a**,**b**) HadCRUT4; (**c**,**d**) GISTEMP; (**e**,**f**) ERA-40. Trends in: (**a**,**c**,**e**) AR(1) and (**b**,**d**,**f**) standard deviation, measured as Kendall τ values. Significance at the 90% confidence interval relative to a null model (see Methods) is indicated with cross-hatching. Maps were created using NCAR Command Language (Version 6.2.1) [Software]. (2014). Boulder, Colorado: UCAR/NCAR/CISL/TDD. http://dx.doi.org/10.5065/D6WD3XH5.

Examining the strength of the autocorrelation and variance trends in each dataset (Fig. 5), the strongest increasing trends in variance and autocorrelation (i.e. ‘slowing down’) are typically around 30–40°N in the North Pacific, in a SW-NE band across the North Atlantic, in a SW-NE band across North America, and in the central Mediterranean. The North Pacific and North Atlantic spatial signals are broadly consistent with the increasing autocorrelation and variance trends seen in the PDO, AMO and Atlantic Tripole indices. Tests for significance of the spatial trends (see Methods) suggest they are significant at the 90% confidence level in many places, including the increasing autocorrelation and variance across SW-NE North America and in the Mediterranean.

We also analysed HadCRUT4, GISTEMP^19^ and the ERA-Interim^20^ reanalysis over its interval 1979–2015. The spatial results (Fig. S10) show an overall shift toward more negative trends in autocorrelation and variance compared to the earlier overlapping interval (1957–2002), although some regions, e.g. in the NE Pacific, show strong increases in both autocorrelation and variance. Such (multi)decadal variability in autocorrelation and variance trends, particularly in the North Atlantic sector, is consistent with the behaviour seen in aggregate climate indices (Figs 2 and S8). The persistence of positive autocorrelation and variance trends in the North Pacific region is also consistent with results for the PDO index (Fig. S1) and previous work^7^.

## Discussion

The very limited ‘memory’ of the atmosphere (e.g. limited heat capacity of the planetary boundary layer) and the limited memory of the land surface (linked to its water holding capacity) suggest that much of the persistence of inter-monthly temperature fluctuations over land must be linked to the large heat capacity, long-timescale dynamics, and associated long memory of the ocean^21^. Much previous work has linked the sign and magnitude of SST fluctuations in particular regions of the ocean to the incidence and magnitude of extreme events on land, including meteorological drought^22, 23^. Where such remote causal teleconnections have been established, one might intuitively expect that the persistence of SST fluctuations in the relevant ocean regions would influence the persistence of tele-connected (causally linked) extreme events on land. Furthermore, we may expect the amplitude and duration of extreme events to be positively correlated, as is seen for El Niño and La Niña events^24^. Having detected positive trends in autocorrelation and variance in SST fluctuations and corresponding climate indices over 1957–2002, we suggest that these are linked to detected positive trends in autocorrelation and variance in land temperatures in regions where there are established spatial correlations and associated mechanistic ocean-land teleconnections. In particular we focus on the North Pacific and North Atlantic ocean regions and their influence on the North American and Mediterranean land regions. First we discuss the ocean regions before turning to the land regions.

Our results show that monthly temperature variability and autocorrelation increased over 1957–2002 across large parts of the North Pacific and North Atlantic, and in the corresponding PDO and AMO indices. Spatially, where the trends in autocorrelation and variance have the same sign this can be interpreted as a change in system timescale (either ‘slowing down’ or ‘speeding up’). Where they have opposing signs alternative explanations are required, for example changes in ocean mixed layer depth and associated heat capacity may be expected to cause opposing changes in autocorrelation and variance^7, 25, 26^. We find evidence of slowing down of SST variability over 1957–2002 across large parts of the North Pacific and North Atlantic, which could be described as a loss of resilience (a weakening of restoring negative feedbacks). More recently, these autocorrelation and variance trends have weakened or reversed in the North Atlantic, whereas they are more consistently positive over time in the North Pacific.

Turning to the North American land region, previous work has linked SST variability including ENSO, the PDO and AMO to the spatial pattern and frequency of drought conditions across North America^23, 27, 28^. For example, multiyear droughts in the 1950s and at the turn of the 21^st^ century have been attributed to SST forcing^28^ and since the mid-1990s shifts in the PDO and AMO to a cold tropical Pacific-warm Atlantic have produced “ideal” conditions for North American drought^23, 28^. Our analysis of 1957–2002 shows increasing SST variance in the tropical North Atlantic and large parts of the North Pacific, and increases in autocorrelation and variance in the PDO and AMO indices. We hypothesise that these trends were at least partly responsible for observed increases in autocorrelation and variance of temperature fluctuations in parts of North America. This increased variance and persistence of land temperature fluctuations can in turn be linked to the persistence and extremity of droughts at the turn of the 21^st^ century, including in the southern Great Plains and in southwest North America. However, it should be noted that over 1979–2015 the autocorrelation and variance trends are much weaker (Fig. S10), yet the southwest North American drought is ongoing, especially in California. This can happen because SST forcing only explains a fraction of Californian winter precipitation variance, with internal atmospheric variability also playing a major role^29^. A further contributing factor may be anthropogenic forcing and associated record high temperatures, which are now playing a greater role in surface moisture deficits in the 2011–2014 Californian drought than previous drought episodes^29, 30^.

Turning to the Mediterranean, previous work has linked decadal variability in the AMO and NAO to decadal climate anomalies^31^. Mediterranean SST variability and summer (but not winter) land surface air temperature anomalies are strongly correlated with AMO variability^31^ over 1960–2000 with positive AMO associated with warmer than usual Mediterranean summers. Warm temperature anomalies in the Indian Ocean also promote Mediterranean drying, and we find evidence for increased autocorrelation and variance of SST anomalies in the Arabian Sea over 1957–2002 (Figs 4 and 5). Hence we suggest that increasing persistence and variance of SST fluctuations in neighbouring parts of the North Atlantic, in the corresponding AMO and Atlantic Tripole indices, and in the Arabian Sea, contributed to observed increases in autocorrelation and variance of Mediterranean temperatures over 1957–2002 (Figs 4 and 5). This increased persistence of Mediterranean temperature fluctuations can in turn be linked to an observed increase in frequency^32^ and extremity^33^ of Mediterranean drought (noting that an anthropogenic forcing contribution to Mediterranean drought is also detectable)^32^.

Although existing models fail to show an anthropogenic forcing component to historical trends in autocorrelation and variance of monthly temperature anomalies, a much stronger future anthropogenic forcing is expected to produce directional trends. For example, Arctic amplification of warming and the projected loss of Arctic sea-ice have been linked to a general decline in temperature variability in the Northern Hemisphere high- and mid-latitudes^13, 34–37^ (although there are some transient, seasonal or regional increases in variability)^13, 34–37^. Interestingly, under high-end future forcing, several of the CMIP5 models show a ‘speeding up’ and drop in amplitude of Atlantic Meridional Overturning Circulation (AMOC) variability^38, 39^, which would be expected to contribute to a drop in autocorrelation and variance of the AMO index. With this we may expect a reduction in persistence and variance of land temperatures in regions strongly influence by the AMO. Conceivably other aspects of the climate system will show increased autocorrelation and variance in a warming world. Thus, to understand how climate variability and persistence may change in a warmer world, we need to better understand not just how the ocean affects climatic variability on parts of the land surface, but also how global warming will affect the internal modes of variability governed by the ocean (the ‘decadal oscillators’).

To conclude, we find significant historical trends in surface ocean memory and variance which appear to be influenced by intrinsic (multi)decadal fluctuations in the climate system. We also find evidence that the changing persistence and variance of sea surface temperature fluctuations contributes to the extremity and persistence of seasonal temperature anomalies on parts of the world’s land surface. Based on a review of established spatial correlations and corresponding mechanistic ‘teleconnections’ we suggest that observed slowing down of North Pacific and North Atlantic sea surface temperature variability contributed to observed increases in land temperature variability and autocorrelation, which in turn contributed to persistent droughts in North America and the Mediterranean.

## Methods

### Datasets and pre-processing

We obtained four climate indices from the NOAA Earth System Research Laboratory (http://www.esrl.noaa.gov/psd/data/climateindices/): Pacific Decadal Oscillation (PDO, 1948–2016), Atlantic Multidecadal Oscillation unsmoothed (AMO, 1948–2015), Atlantic Tripole EOF (Atlantic Tripole, 1948–2008), North Atlantic Oscillation (NAO, 1950–2016). We restricted the analysis presented in the main paper to the ERA-40 time period 09/1957-08/2002. The Atlantic Tripole EOF is calculated as the 1st Empirical Orthogonal Function (EOF) of the Sea Surface Temperatures (SST) located at 10N–70°N and 0–80°W. All these records are based on instrumental observations and not on reconstructions. We used the raw data without any pre-processing for the calculation of autocorrelation and variance trends.

We considered the HadCRUT4^18^ (http://www.metoffice.gov.uk/hadobs/hadcrut4/), GISTEMP^19^ (http://data.giss.nasa.gov/gistemp/), and the ERA-40^16^/ERA-Interim^20^ reanalysis temperature datasets. We focused on the ERA-40 time period 09/1957–08/2002 (ERA-40, HadCRUT4, GISTEMP) but also considered the ERA-Interim time period 01/1979–08/2015 (ERA-Interim, HadCRUT4, GISTEMP) in the Supplementary Information. All time series are of monthly resolution and the seasonal cycle has been removed. HadCRUT4 and GISTEMP already come in anomalies relative to a mean seasonal cycle in a base period (1961–90 for HadCRUT4 and 1951–1980 for GISTEMP). Unfortunately this procedure is known to bias the estimates because of the difference in the true mean and variance and the sample from the base period^15^. The bias is most likely not too large for qualitative results, but it is an argument to compare different datasets. ERA reanalysis datasets and the climate models do not have this problem. There, we removed the annual cycle in a sliding window (of the same length – 10 years – as used for the bandwidth in the subsequent analysis).

We considered modelled AMO and PDO indices in the historically-forced runs of 9 models (listed in Table 2) from the CMIP5 database. For comparability we construct these indices using the same procedure used to derive observed indices: First, we select the time period under consideration and the region relevant for each index (100–260°E, 20–70°N for the PDO, 80°W-0°, 0–60°N for the AMO; land grid cells are not considered, nor are those containing sea ice at any time). To remove the trend induced by anthropogenic warming we subtract the global mean sea surface temperature at each time point. We then calculate monthly anomalies by subtracting the mean annual cycle at each grid cell separately. The PDO then follows as the coefficient of the first empirical orthogonal function (principle component) of the anomalies’ variability pattern, calculated from their covariance matrix^40^. The AMO is simply the spatial mean of the region under consideration.

### Calculation of autocorrelation and variance trends

All calculations were performed on residuals after each record was detrended by applying a Gaussian kernel function with a prescribed bandwidth. We estimated autocorrelation at lag 1 (AR1) and variance (measured as standard deviation, SD) within a sliding window that we moved one point ahead (1 month) along each record. As default for the spatial temperature datasets, we used a detrending bandwidth of 10 years and a sliding window of 25 years. For the indices, we used a range of values for bandwidth and window size (see Sensitivity Analysis). We calculated the trend in the obtained AR1 and SD values based on the Kendal *τ* rank correlation coefficient. A positive Kendal *τ* signals increasing trends, while a negative decreasing trends in the indicators. For example, a monotonous increase over time would yield *τ* = 1, while a monotonous decrease would yield *τ* = −1; no trend would yield *τ* = 0.

### Sensitivity analysis

We explored the sensitivity of the Kendal *τ* trends to the filtering and sliding window parameter choices by estimating trends for both AR1 and SD for a combination of sliding window sizes and filtering bandwidths. In particular, we used a minimum sliding window size of 5 years that we increased in increments of 5 years up to a maximum window size equal to 75% of the record size (e.g. 35 years for the period 1957–2002). We used eight different bandwidths (*h* = 0.5, 1, 2.5, 5, 10, 15, 20, and 30 years) for the Gaussian kernel filter. We also estimated trends without filtering (*h* = 0).

### Significance testing

We compared our empirical Kendal τ trends to trends expected based on a stationary null model. Our null model first generated 1000 surrogate records with the same Fourier spectrum and amplitudes as the original records^41^. For each surrogate record, we estimated Kendal τ trends for the same combination of sliding window size and filtering bandwidth that we used in the original records. Lastly, for each of these combinations, we compared the occurrence of the empirical Kendal τ to the null distribution based on the surrogates. Occurrences below 5% (*p* = 0.1 two tailed) were considered significant. We reported the fraction of the combinations of sliding window size and filtering bandwidth where the trend was significant.

### Robustness analysis

We tested how robust were the Kendal τ trends when estimated at different periods within the empirical records. To do this we picked a size of 22.5 years (half the total period of the 1957–2002 records). We then selected periods of this size at all possible positions along the record. For all of these periods, we then analyzed trends in AR1 and SD using a sliding window half the size of the period (11.25 years). The records were filtered with a Gaussian smooth function of 11.25 years bandwidth.

All analyses were performed in R (v3.2.0) using modified functions from the *earlywarnings* package (https://github.com/earlywarningtoolbox) following the methodology described in ref. 42.

## Electronic supplementary material

Supplementary Information
