# Supplementary material for: Observed trends in the magnitude and persistence of monthly temperature variability
Source: Sci Rep. 2017 Jul 19;7:5940. doi: 10.1038/s41598-017-06382-x (PMC5517648; doi:10.1038/s41598-017-06382-x)
Supplement: Supplementary file 1 — Supplementary Information [file 41598_2017_6382_MOESM1_ESM.pdf]

Supplementary Information for:

Observed trends in the magnitude and persistence of monthly temperature variability

Timothy M. Lenton[1]*, Vasilis Dakos[2,3], Sebastian Bathiany[4] and Marten Scheffer[4]

[1]Earth System Science Group, College of Life and Environmental Sciences, University of Exeter, Exeter EX4 4QE, UK.

[2]Institute of Integrative Biology, Center for Adaptation to a Changing Environment, ETH Zurich, Switzerland.

[3]Institut des Sciences de l'Evolution de Montpellier (ISEM), BioDICe team, CNRS, Universite de Montpellier, Montpellier, France.

[4]Department of Environmental Sciences, Wageningen University, P.O. Box 47, NL-6700 AA, Wageningen, The Netherlands.

*e-mail: t.m.lenton@exeter.ac.uk

Supplementary Figures

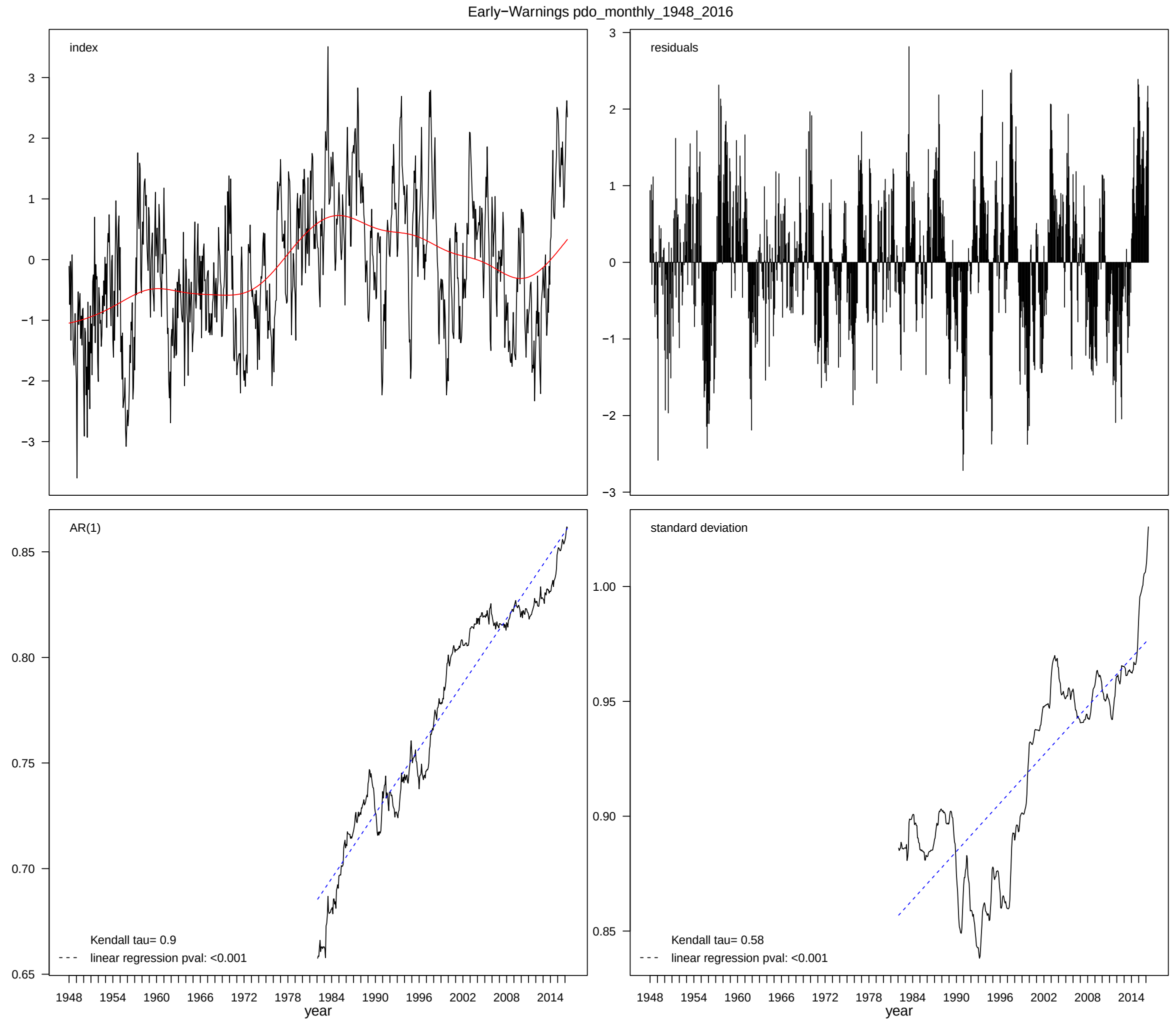

Figure S1. Trends in autocorrelation and variance in the monthly PDO index 1948-2016. Top left panel: original climate index (red line is the Gaussian smoothing function with a bandwidth of 10 years used for filtering). Top right panel: residuals after Gaussian filtering. Bottom panels: autocorrelation at lag 1 (left) and standard deviation (right) estimated within a sliding window length of half the interval.

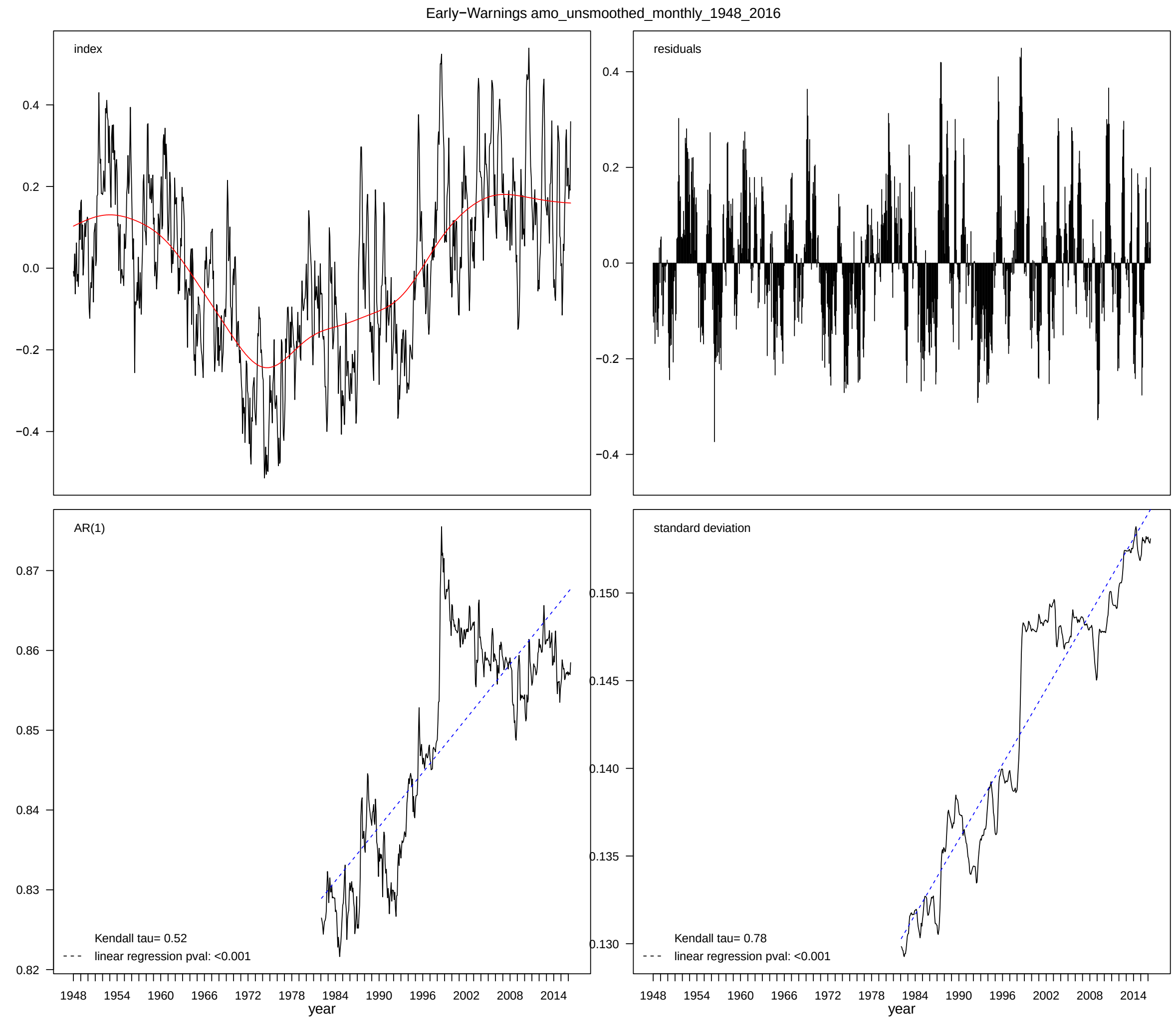

Figure S2. Trends in autocorrelation and variance in the monthly AMO index 1948-2016. Top left panel: original climate index (red line is the Gaussian smoothing function with a bandwidth of 10 years used for filtering). Top right panel: residuals after Gaussian filtering. Bottom panels: autocorrelation at lag 1 (left) and standard deviation (right) estimated within a sliding window length of half the interval.

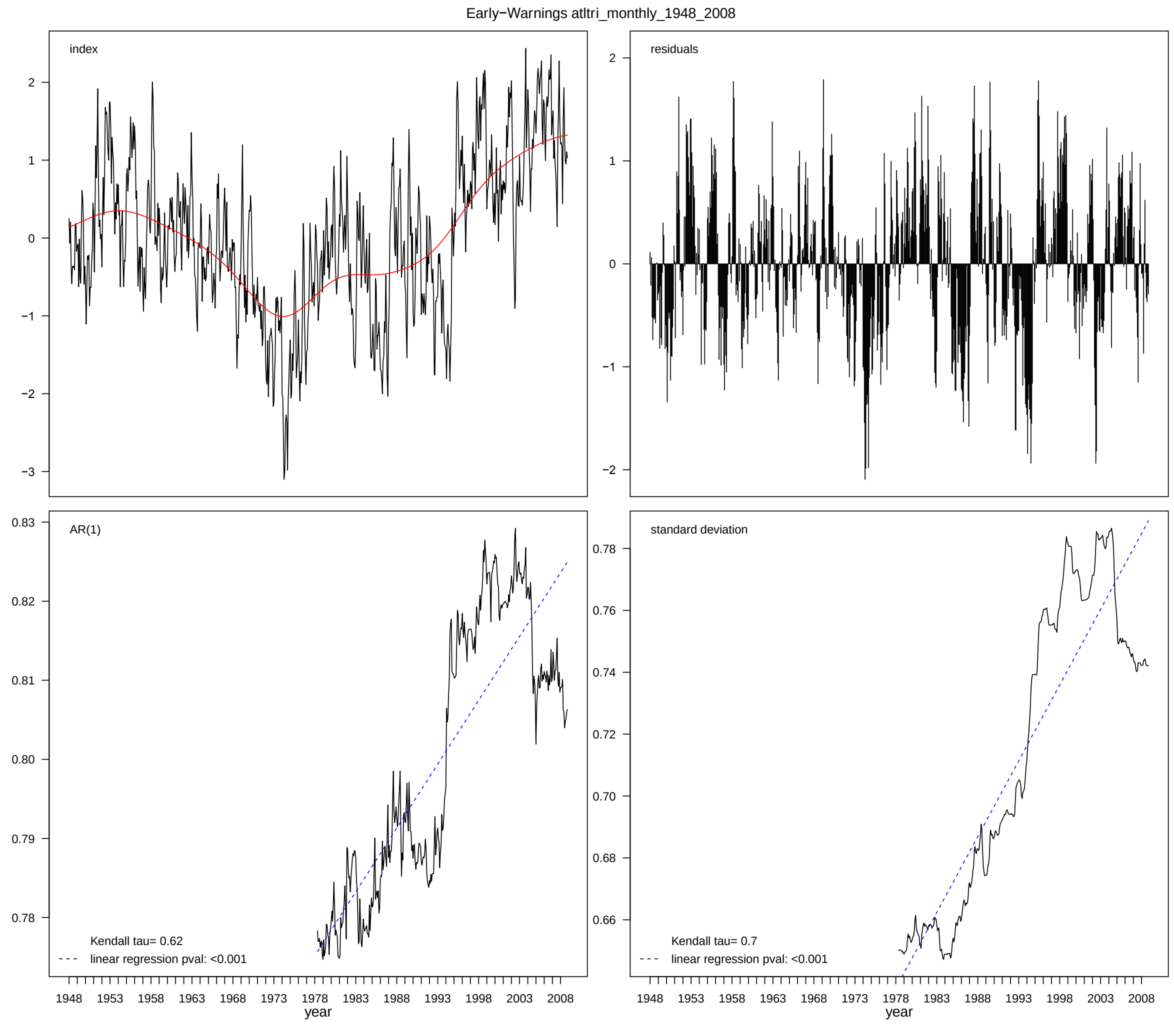

Figure S3. Trends in autocorrelation and variance in the monthly Atlantic Tripole index 1948-2008. Top left panel: original climate index (red line is the Gaussian smoothing function with a bandwidth of 10 years used for filtering). Top right panel: residuals after Gaussian filtering. Bottom panels: autocorrelation at lag 1 (left) and standard deviation (right) estimated within a sliding window length of half the interval.

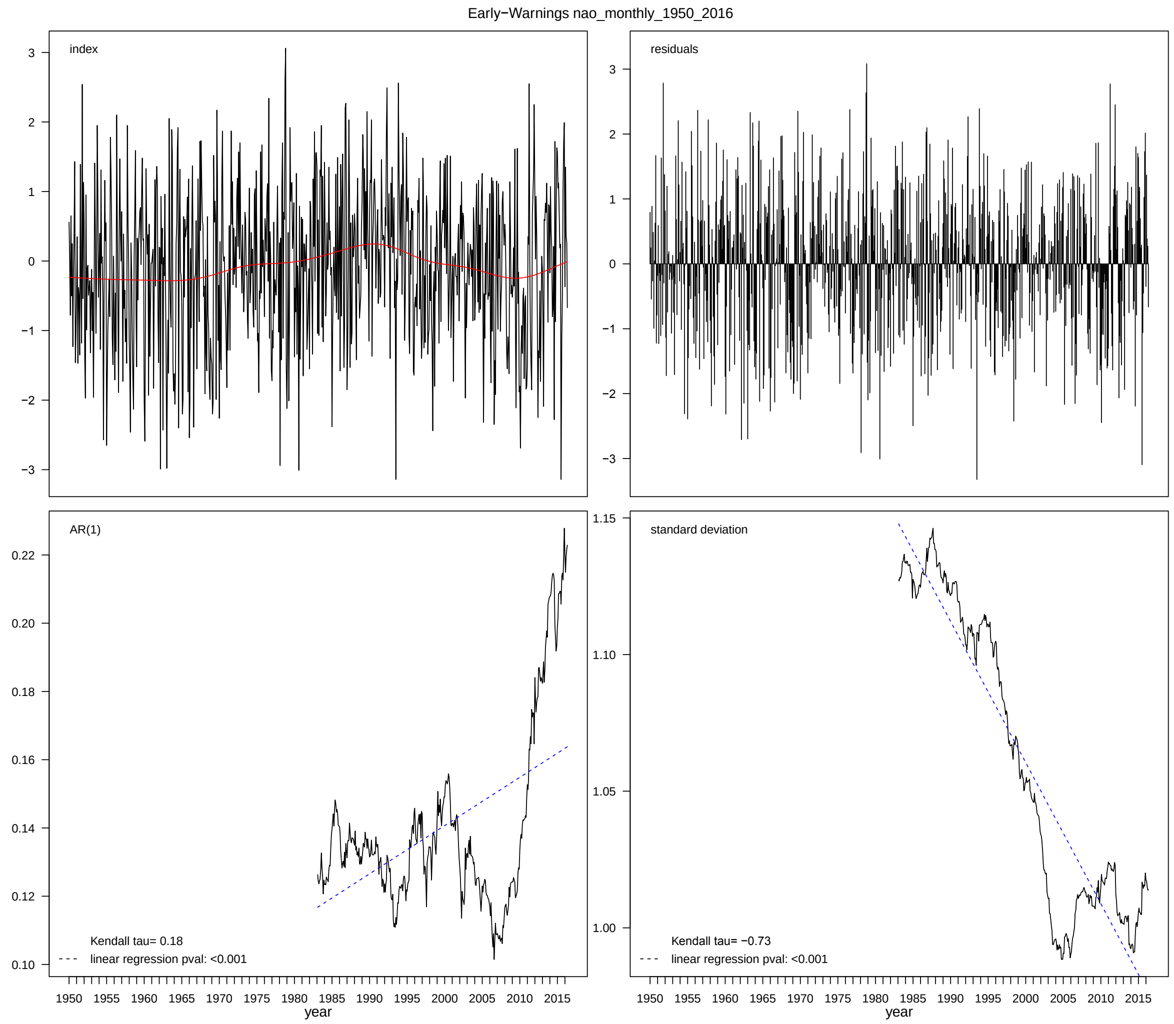

Figure S4. Trends in autocorrelation and variance in the monthly NAO index 1950-2016. Top left panel: original climate index (red line is the Gaussian smoothing function with a bandwidth of 10 years used for filtering). Top right panel: residuals after Gaussian filtering. Bottom panels: autocorrelation at lag 1 (left) and standard deviation (right) estimated within a sliding window length of half the interval.

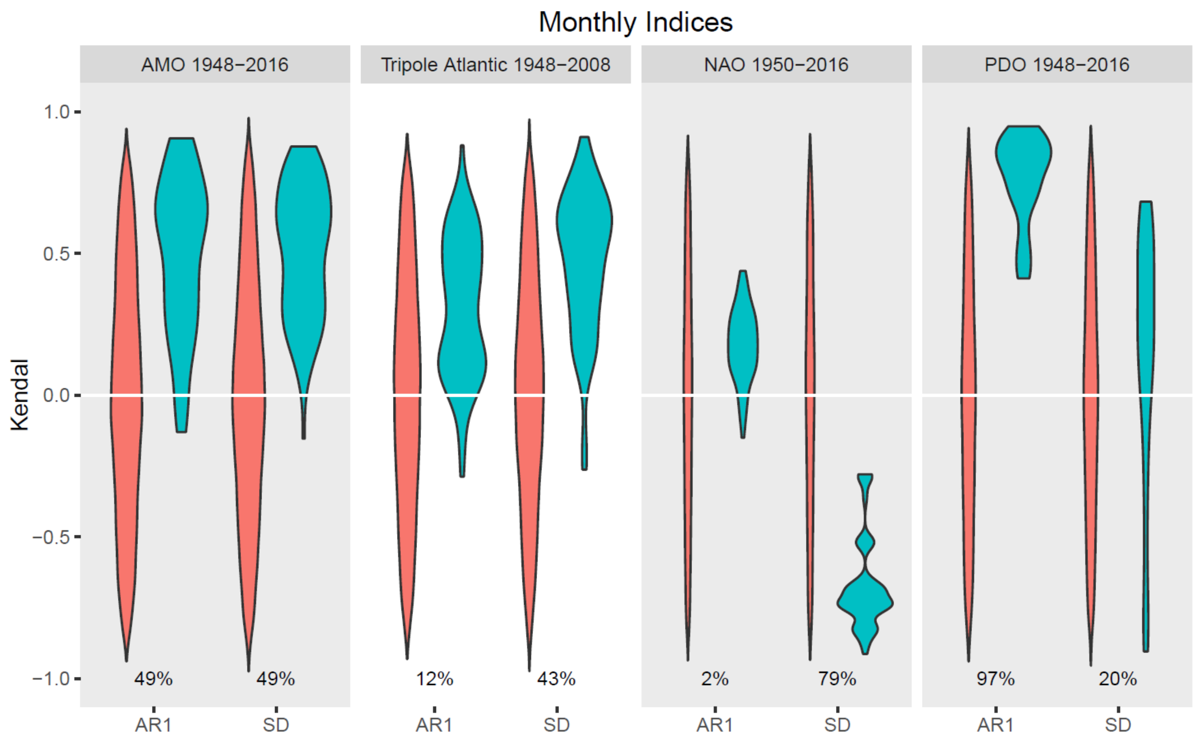

Figure S5. Autocorrelation and variance trends in climate indices across their original time intervals. For the full temporal range of each of the NOAA indices. Distribution of Kendall τ trends in AR(1) and standard deviation for all combinations of sliding window and bandwidth size in observational climate indices (green) and null models (red; from 1000 time-series with same frequency spectrum) (see Methods sections Sensitivity, Significance). The percentages represent the fraction of results from the observational indices that are significantly different to the null models (p = 0.1 two tailed).

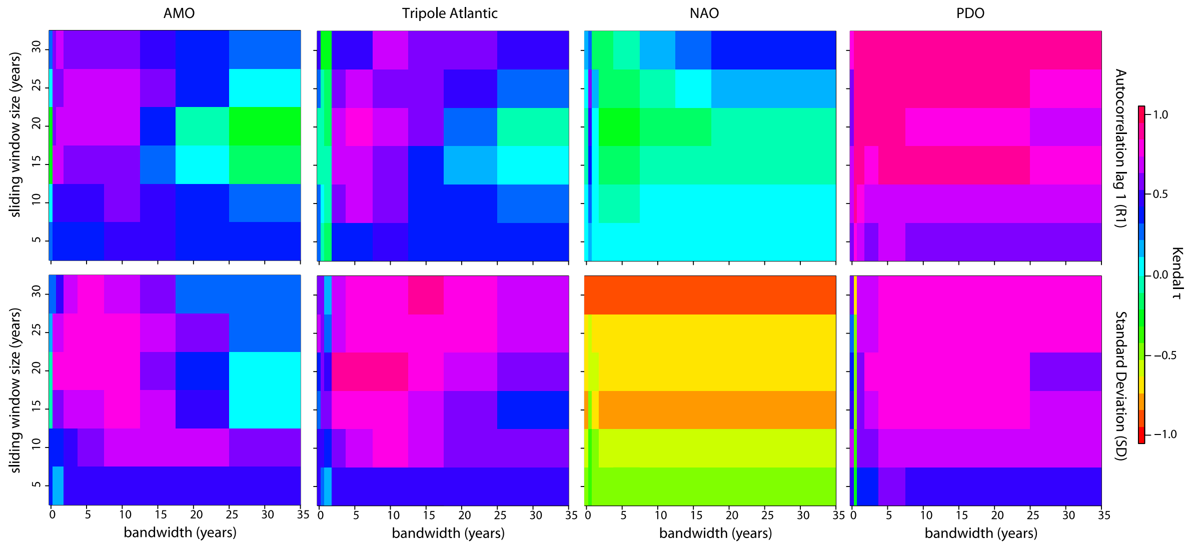

Figure S6. Sensitivity analyses varying the filtering bandwidth and sliding window length. Results for the observational indices over the ERA-40 interval (1957-2002): (top) AR1, (bottom) SD.

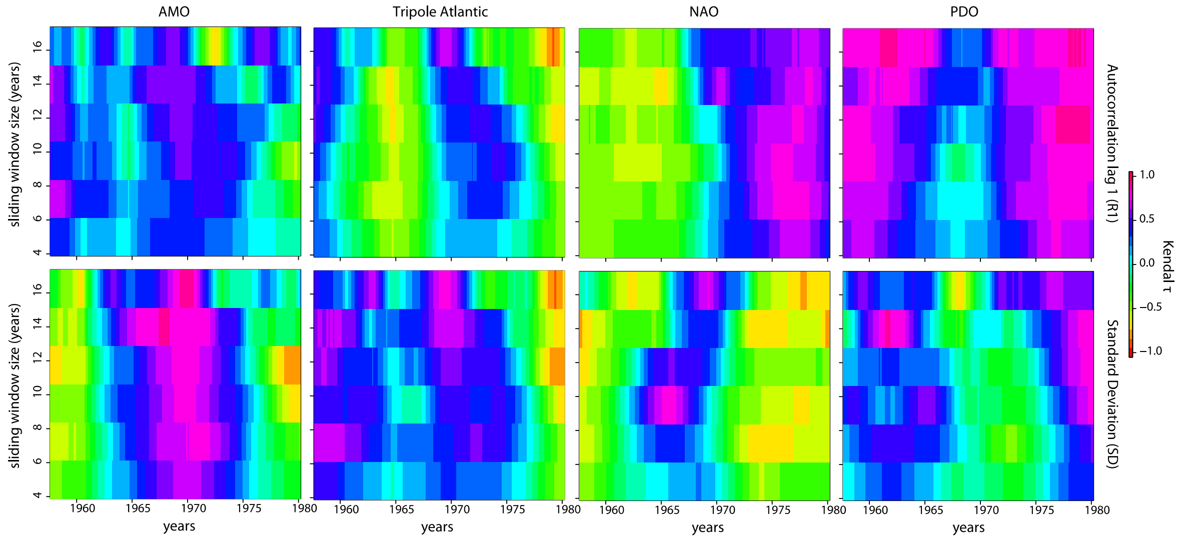

Figure S7. Robustness analyses varying the 22.5 year period of analysis. Results for the observational indices over the ERA-40 interval (1957-2002) using filtering bandwidth 11 years 3 months and varying sliding window length: (top) AR1, (bottom) SD.

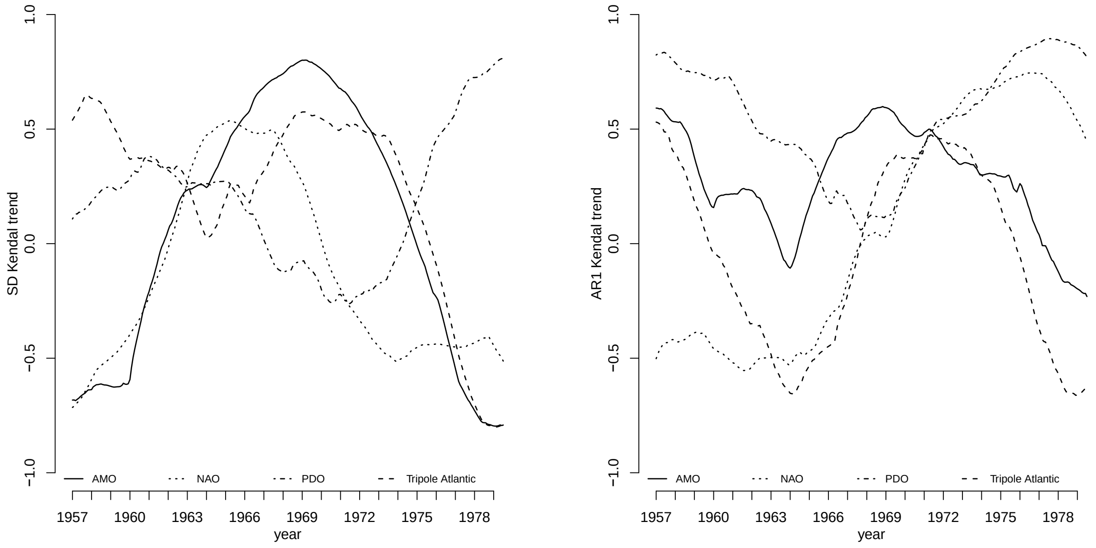

Figure S8. Decadal variability in variance and autocorrelation trends in climate indices. Results for the AMO, NAO, PDO and Atlantic Tripole, in each case for 22.5 year intervals (within 1957-2002) analysed with filtering bandwidth and sliding window length of half the interval (11 years 3 months). (left) Standard Deviation trends in the four climate indices, (right) Autocorrelation trends.

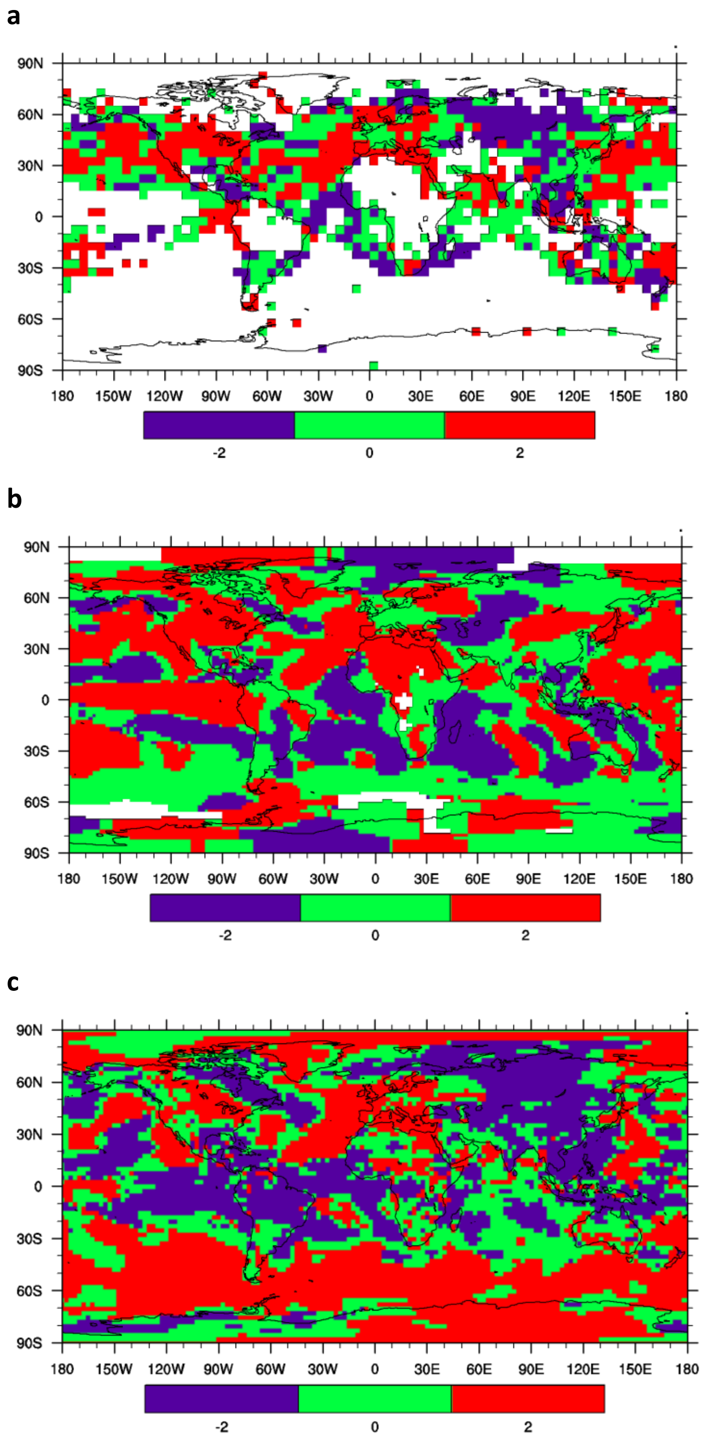

Figure S9. Consistency between autocorrelation and variance trends within temperature datasets. Monthly temperature datasets for the interval 1957-2002, processed with filtering bandwidth 10 years and sliding window length 25 years: **a.** HadCRUT4. **b.** GISTEMP. **c.** ERA-40. Red (2) indicates regions where both AR(1) and SD have a positive trend, dark blue (-2) indicates regions where both AR(1) and SD have a negative trend, green (0) indicates regions where they have opposing trends. Maps were created using NCAR Command Language (Version 6.2.1) [Software]. (2014). Boulder, Colorado: UCAR/NCAR/CISL/TDD. http://dx.doi.org/10.5065/D6WD3XH5.

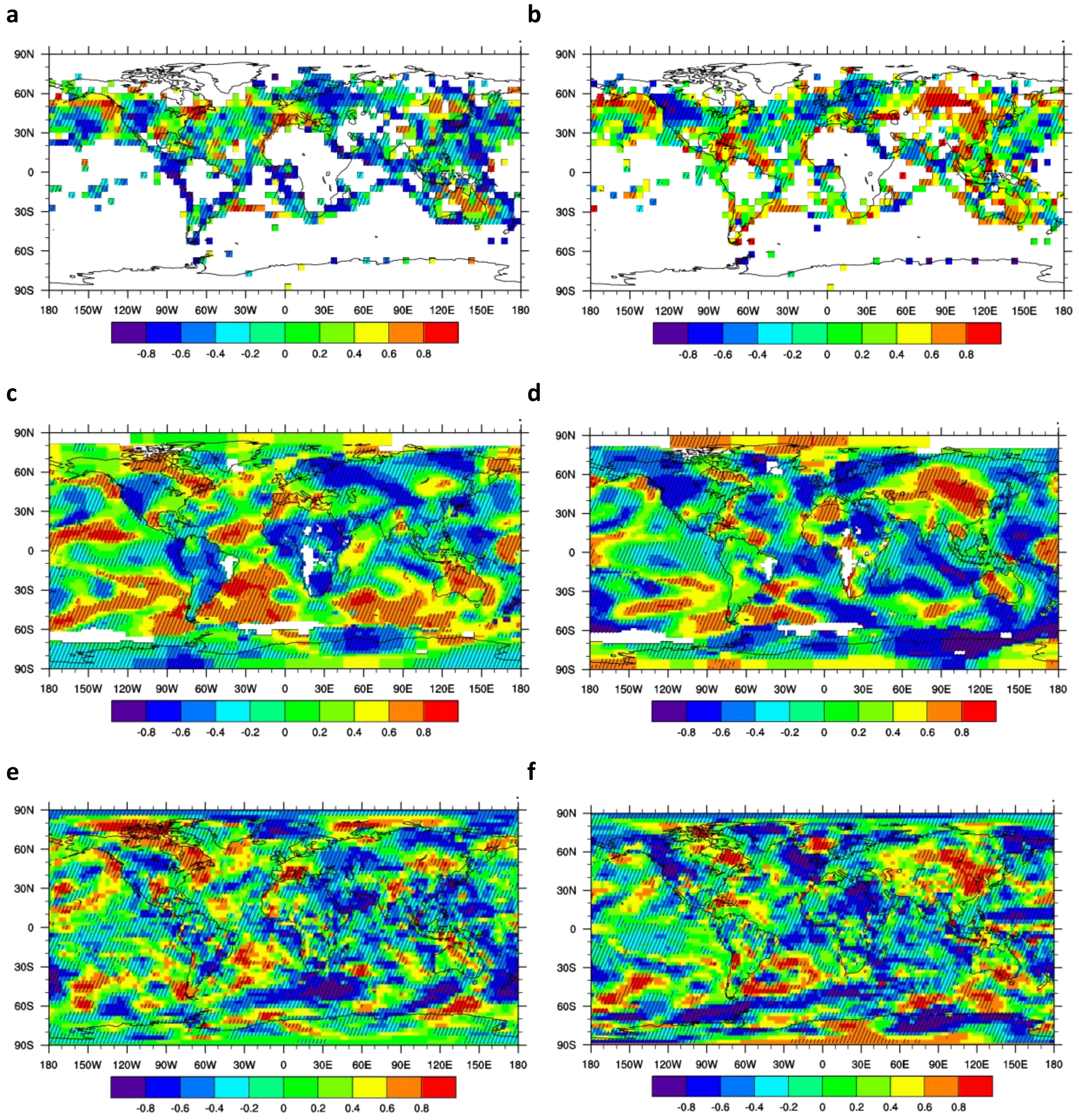

Figure S10. Trends in autocorrelation and variance in different temperature datasets 1979-2015. Monthly temperature datasets processed with filtering bandwidth 10 years and sliding window length 15 years: **a,b.** HadCRUT4; **c,d.** GISTEMP; **e,f.** ERA-Interim. Trends in: **a,c,e.** AR(1) and **b,d,f.** standard deviation, measured as Kendall τ values. Significance at the 90% confidence interval relative to a null model (see Methods) is indicated with cross-hatching. Maps were created using NCAR Command Language (Version 6.2.1) [Software]. (2014). Boulder, Colorado: UCAR/NCAR/CISL/TDD. http://dx.doi.org/10.5065/D6WD3XH5.
